# Identity and relationships of the Arboreal Caatinga among other floristic units of seasonally dry tropical forests (SDTFs) of north-eastern and Central Brazil

**DOI:** 10.1002/ece3.91

**Published:** 2012-02

**Authors:** Rubens M Santos, Ary T Oliveira-Filho, Pedro V Eisenlohr, Luciano P Queiroz, Domingos B O S Cardoso, Maria J N Rodal

**Affiliations:** 1 Departamento de Ciências Florestais, Universidade Federal de Lavras37200-000, Lavras, MG, Brazil; 2 Departamento de Botânica, Instituto de Ciências Biológicas, Universidade Federal de Minas Gerais31270-901, Belo Horizonte, MG, Brazil; 3 Departamento de Biologia Vegetal, Universidade Estadual de Campinas13083-862, Campinas, SP, Brazil; 4 Departamento de Ciências Biológicas, Universidade Estadual de Feira de Santana44036-900, Feira de Santana, BA, Brazil; 5 Departamento de Biologia, Universidade Federal Rural de Pernambuco52171-900, Recife, PE, Brazil

**Keywords:** Floristic connections, geo-climatic variables, seasonally dry tropical vegetation, spatial distribution patterns, tree species composition, vegetation–environment relationships

## Abstract

The tree species composition of seasonally dry tropical forests (SDTF) in north-eastern and central Brazil is analyzed to address the following hypotheses: (1) variations in species composition are related to both environment (climate and substrate) and spatial proximity; (2) SDTF floristic units may be recognized based on peculiar composition and environment; and (3) the Arboreal Caatinga, a deciduous forest occurring along the hinterland borders of the Caatinga Domain, is one of these units and its flora is more strongly related to the *caatinga* vegetation than to outlying forests. The study region is framed by the Brazilian coastline, 50th meridian west and 21st parallel south, including the Caatinga Domain and extensions into the Atlantic Forest and Cerrado Domains. Multivariate and geostatistic analyses were performed on a database containing 16,226 occurrence records of 1332 tree species in 187 georeferenced SDTF areas and respective environmental variables. Tree species composition varied significantly with both environmental variables and spatial proximity. Eight SDTF floristic units were recognized in the region, including the Arboreal Caatinga. In terms of species composition, its tree flora showed a stronger link with that of the Cerrado Dry Forest Enclaves. On the other hand, in terms of species frequency across sample areas, the links were stronger with two other units: Rock Outcrops Caatinga and Agreste and Brejo Dry Forests. There is a role for niche-based control of tree species composition across the SDTFs of the region determined primarily by the availability of ground water across time and secondarily by the amount of soil mineral nutrients. Spatial proximity also contributes significantly to the floristic cohesion of SDTF units suggesting a highly dispersal-limited tree flora. These units should be given the status of eco-regions to help driving the conservation policy regarding the protection of their biodiversity.

## Introduction

Seasonally dry tropical forests (SDTFs) have been recently proposed as one of the world's main biomes, or global metacommunity, of tropical vegetation associated with erratic water availability and, in most cases, mineral-rich substrates (Pennington et al. 2009). They are found across a wide area in the Neotropics that, in rough terms, encircles the Amazon basin, in South America, and extends north toward Mexico and the Caribbean (Linares-Palomino et al. 2011). Despite their wide distribution in South America, most SDTFs occur as isolated patches (“nuclei”) and the only vast continuous area is the Caatinga Biogeographic Domain in north-eastern Brazil, with about 800,000 km^2^ (Fernandes 2003), named after its prevalent vegetation type, the *caatinga*, from *caa* ( = forest) and *tinga* ( = white) in the native Tupi language. The *caatinga* is generally described as a woody vegetation with discontinuous canopy, variable in both height (3–9 m) and density, composed mostly of succulent (cacti essentially) and nonsucculent shrubs and trees, most of which are armed with either thorns or prickles and bear microphyllous foliages, though they are leafless during the long-lasting periods of drought; the ground layer is rich in bromeliads, annual herbs, and geophytes (Ab'Sáber 1974; Eiten 1983; Prado 2003; Cardoso and Queiroz 2010). The *caatinga* covers most of the *Depressão Sertaneja*, a great extent of semiarid lowlands (<400 m a.s.l) intervened by emerging tablelands, highland ridges, and sandy deposits, with contrasting climates and vegetation types, including *cerrado* (savanna woodlands), *campos rupestres* (rocky grass- and scrublands), and seasonal deciduous forests (Andrade-Lima 1981; Rodal and Sampaio 2002; Ab'Sáber 2003). Other smaller but still extensive SDTF nuclei in South America include the marginal areas of the Gran Chaco Domain, in Argentina, Bolivia and Paraguay, and the Guajira nucleus, in coastland Colombia and Venezuela (Linares-Palomino et al. 2011). Much smaller SDTF nuclei are scattered in between, as those lodged within rain-shadowed inter-Andean dry valleys in Ecuador, Peru, and Bolivia (Killeen et al. 2006; Linares-Palomino 2006; Wood 2006), and those embedded in the *cerrado* vegetation of Central Brazil on patches of exceptionally fertile soils (Oliveira-Filho and Ratter 2002).

**Figure 1:**
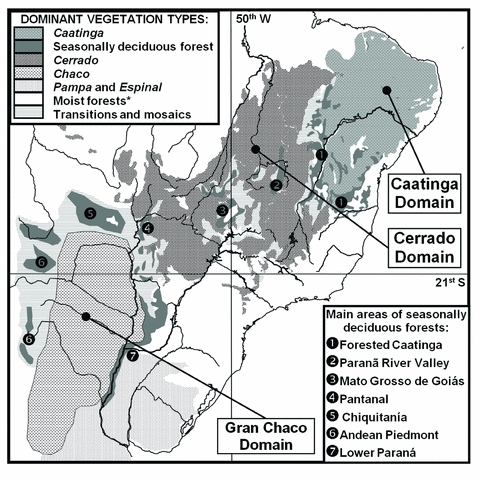
The three main Phytogeographic Domains of South America that form the “diagonal of open formations” and are named after their dominant vegetation types, and location of seven main extents of seasonally deciduous forests either outside or adjacent to the Caatinga Domain. (*Moist forests = evergreen rain forests + seasonal semideciduous forests).

SDTFs are presently a growing focus of attention because they are highly endangered and poorly known (Mooney et al. 1995; Miles et al. 2006; Pennington et al. 2006; Werneck 2011). A number of studies are based on the hypothesis that SDTFs had expanded and merged during the drier glacial periods of the Quaternary and contracted and fragmented during the moister interglacial periods (Pennington et al. 2000). Because of its circum-Amazonian configuration, this hypothetical plant evolution theatre in South American was referred to as “Pleistocenic Arc” by Prado and Gibbs (1993). In this seminal paper, the authors assessed the hypothesis of past connections between the *caatinga* and *chaco* vegetation, the two semiarid extremes of the so-called northeast –southwest “diagonal of open formations” that includes the *cerrado* in between (Fig. 1). The hypothesis was then based on similar vegetation physiognomy (predominance of deciduous, sclerophyllous, and succulent plants) and the abundance of species of Cactaceae, Fabaceae, Capparaceae, Anacardiaceae, and Nyctaginaceae (Rizzini 1963, 1979; Cabrera 1971; Bigarella et al. 1975; Andrade-Lima 1957, 1981; Cabrera and Willink 1980), though Fernandes and Bezerra (1990) opposed it, asserting a strong dissimilarity at the species and genus levels. Prado and Gibbs (1993) scrutinized a suite of widespread species seeking for those shared by the two vegetation regions but found instead a strong connection among the floras of the *caatinga* and that of the deciduous dry forests of both the rich soil enclaves of the Cerrado Domain and the outskirts of the Gran Chaco Domain, particularly the Bolivian Chiquitano. Since then, this pattern has been strongly confirmed (e.g., Fernandes 1998; Prado 2003; Oliveira-Filho et al. 2006; Zanella 2010), reinforcing the exclusion of the core *chaco* flora from the SDTF concept (Pennington et al. 2000, 2009), and eventually contributing to consolidate SDTF as an ecologically meaningful concept.

Attempts to extend the floristic links among SDTFs across a wider range in the Neotropics were much less successful because there seems to be a considerable floristic disruption beyond the Caatinga-Chiquitano axis as demonstrated by Linares-Palomino et al. (2011) in the first overall analyses of the species composition of predefined Neotropical SDTF nuclei. The authors distinguished four main floristic groups, with those of the Caatinga-Chiquitano axis composing the “Brazilian Group.” The analyses also indicated that the Brazilian Group is probably composed of a larger number of SDTF “floristic units” (used hereafter to contrast with the wider concept of “nuclei,” or the even wider “Group”) than previously thought, as already demonstrated for the Caatinga Nuclei by Queiroz (2006), based on the distribution of legume species. Detailed floristic analyses are then necessary to clarify the floristic heterogeneity within the Brazilian Group.

The seasonal deciduous forests occurring within and around the Caatinga Domain, in particular, are far from any consensus regarding their delimitation, nomenclature, and classification, and the main reason for this stems from their high heterogeneity, apparently related to variations in climate, topography, and substrate (Rodal et al. 2008). Some authors opted to include them in a single wide group and related their distribution to transitional areas where the semiaridity is replaced by strongly seasonal climates (Veloso et al. 1991). These shifts take place in three main circumstances. The first includes the so-called *matas de brejo*, which are forest enclaves occurring on hinterland highlands where the rainfall is locally increased by the forced uplift of air masses (Rodal 2002). The other two are related to the transitional climates of the marginal areas of the Caatinga Domain contacting either the Cerrado or the Atlantic Forest Domains (Sampaio et al. 1981). The eastern transition to the Atlantic forests used to be a narrow stretch of deciduous forests, known as *agreste*, which have been almost completely replaced by croplands (Melo and Rodal 2003).

The transition to the Cerrado Domain stretches along the southern and western boundaries of the Caatinga Domain, in northern Minas Gerais state and southwestern Bahia state, and is predominantly covered by a comparatively wider stretch of deciduous forests with a controversial identity and classification although *caatinga arbórea*, hereafter “Arboreal Caatinga,” is traditionally used by most experts (e.g., Fernandes 1998; Taylor and Zappi 2004; Cardoso and Queiroz 2010). Andrade-Lima (1971, 1981) stated that, despite the remarkable physiognomic difference between the typical *caatinga* and the Arboreal Caatinga (e.g., taller trees and scantier succulents in the latter), the long leafless period and, above all, the floristic composition are strong arguments in favor of including this vegetation in the Caatinga Domain. On the other hand, some authors and the Brazilian Government have treated the Arboreal Caatinga as a hinterland extension of the Atlantic Forest Domain/Biome (e.g., Silva and Casteleti 2005; Oliveira-Filho et al. 2006; IBGE 2008). In addition, the indiscriminate use of a second (and hazy) name, *mata seca* ( = dry forest), to refer to either or both the Arboreal Caatinga and the deciduous forest enclaves of the Cerrado Domain has spread great havoc with serious impacts on conservation issues (Oliveira-Filho 2006).

The Arboreal Caatinga, treated as “Peri-Caatinga seasonal forests” by Linares-Palomino et al. (2011), is apparently a consistent unit along others of the Brazilian Group. We here go a step further in data refinement and analysis seeking to clarify the spatial patterns of the SDTF woody flora in a particular frame of the Brazilian Group, including the whole Caatinga Domain and neighboring SDTF areas in north-eastern and Central Brazil. Although our initial purpose was to shed a light on the floristic identity and relationships of the Arboreal Caatinga, we ended up with a wider treatment of SDTF areas in the whole region because the question addressing the Arboreal Caatinga was subordinate to much wider questions.

We then addressed three hypotheses stemming from the literature: (1) variations in species composition among SDTF areas in the region are related to both geo-climatic variables and spatial proximity; (2) SDTF floristic units may be recognized in the region based on coherent and peculiar species composition and geo-climatic features; and (3) the Arboreal Caatinga is one of such units and, as first proposed by Andrade-Lima (1971), its species composition is more strongly related to the *caatinga* vegetation than to outlying deciduous forests.

## Methods

### The dataset of SDTF areas

**Figure 2:**
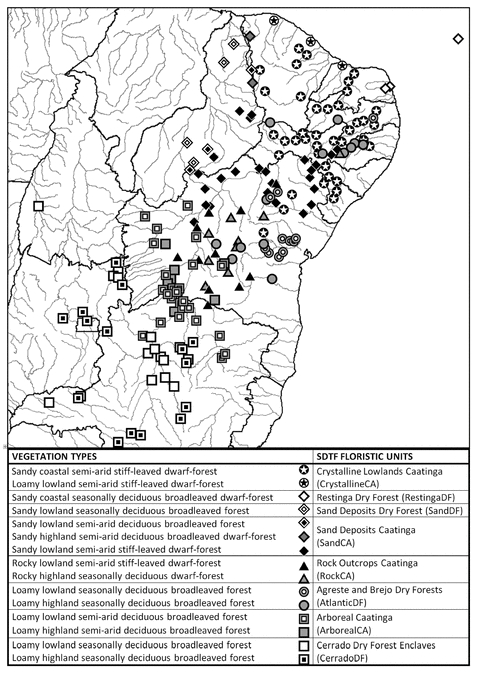
Location of the 187 areas of seasonally dry tropical forests (SDTFs) used in the floristic analyses, and their classification into 15 vegetation types, following Oliveira-Filho (2009), and eventually merged into eight floristic units. Acronyms are given within parentheses.

We extracted the dataset from TreeAtlan 2.0, a relational database compiled from the literature and herbarium specimens that contains tree species occurrence records (checklists), geographic location, vegetation type, and environmental data (geo-climatic variables) for more than 1500 areas in eastern tropical and subtropical South America (see description, history and protocol of TreeAtlan 2.0 at http://www.icb.ufmg.br/treeatlan; extraction dated on March 20, 2011). The dataset consisted of all 187 SDTF areas contained by TreeAtlan 2.0 within the geographic space delimited by the Atlantic Ocean, the 50th meridian west and 21st parallel south (Fig. 2). These limits were established in order to include the whole Caatinga Domain plus large expanses into the neighboring Atlantic Forest and Cerrado Domains.

We organized the dataset into two matrices, both with the 187 SDTF areas as row headings. The species matrix, obtained from the checklists, contained 1332 tree species as column headings and binary occurrence records as entries, of which 16,226 were presence records. The environmental matrix contained 32 variables as column headings. Variables related to location included latitude, longitude, altitude, and shortest distance to the ocean. The vegetation type (Fig. 2) was included as a categorical variable. The classification followed the system proposed by Oliveira-Filho (2009) for the vegetation of eastern tropical and subtropical South America, which is a development of the widely accepted IBGE classification system for Brazilian vegetation (Veloso et al. 1991). This new system seeks to describe physiognomic and environmental variations at much smaller scales than those covered by the IBGE and therefore offers additional and optional descriptive terms.

Four substrate-related variables, also categorical were eventually replaced by ranks (1, 2, 3), or their product. They consisted of (1) soil fertility: 1 = “dystrophic” (saturation of bases < 30%), 2 = “mesotrophic” (30–70%), 3 = “eutrophic” (>70%); (2) soil texture: 1 = “sandy” (sand fraction > 70%), 2 = “sand-loamy” (30–70%), 3 = “loamy” (<30%); (3) soil depth: 1 = “bare rock to shallow” (0–10 cm deep), 2 = “deep to shallow” (10–50 cm), 3 = “deep” (>50 cm); and (4) soil water storage capacity, obtained by the (1) × (2) product. These variables were extracted from a detailed map of soil types produced by Embrapa &IBGE (2003) using the RadamBrasil Soil Survey of the 1970s and 1980s, and additional field studies carried out by both governmental agencies. Climatic variables included the “mean duration of water-deficit periods,” extracted from Walter diagrams (Walter 1985); the 19 bioclimatic variables produced by WorldClim 1.4, a high-resolution (1 km) database of global climate layers created by Hijmans et al. (2005) based on mean monthly temperature and precipitation; and three additional variables derived from WorldClim by Zomer et al. (2007, 2008): the “potential evapotranspiration,” the “aridity index,” and the “extra-terrestrial solar radiation.”

### Multivariate and spatial analyses

In order to reduce the dimensionality of the species matrix into species composition gradients, we made a number of trials with the ordination techniques available in PC-ORD 6 (McCune and Mefford 2011). Because species singletons commonly increase the intrinsic data noise without relevant contribution to ordination patterns (Lepš and Šmilauer 2003), we performed an a priori elimination of 326 species. We then performed an outlier analysis (McCune and Grace 2002) of this matrix and removed a single area (Jacobina) from the species and environmental matrices because of its strong outlier effect. The final matrices ended up with 186 areas and 996 species. The ordination trials included three techniques described in McCune and Grace (2002): Correspondence Analysis, CA; Non-Metric Multidimensional Scaling, NMS; and Detrended Correspondence Analysis, DCA. The CA was disposed of because, unlike the two other techniques, it showed a very strong compression effect. In the end, we chose the DCA and turned down the NMS because the former produced the best results in terms of interpretive power of the species composition gradients (Kent and Coker 1992). Instead of following any line of methodological reasoning, we went for this pragmatic choice because, although some NMS enthusiasts assert that DCA should be discarded for good (e.g., McCune and Grace 2002), others sustain its applicability (e.g., Lepš and Šmilauer 2003) and both techniques are still widely used (von Wehrden et al. 2009). As stated by Wildi (2010), “the quality of the data used is far more important than the selection of the best ordination method.”

After a few trials with DCA, we chose the default options in PC-ORD 6 (26 segments, no down weighting of rare species, and axes rescaled at threshold = 0) because alternative options produced very similar results. We assessed the species replacement along ordination axes through the length of gradients yielded by DCA and the significance of eigenvalues through randomization tests with 1000 permutations (McCune and Mefford 2011). In order to assess the spatial structure of ordination scores, we used the software SAM 4.0 (Rangel et al. 2010) to produce correlograms for each significant DCA axis plotting the Moran's I across default distance classes (Legendre and Fortin 1989; Diniz-Filho et al. 2003).

We then used the ordination scores as response variables representing the main species composition gradients summarized by DCA axes. We then scrutinized the geo-climatic variables to select a subset of predicting variables. To this end, we checked the whole set for multicollinearity using the variance inflation factor (VIF) and a cutoff value of 10 (Seaby and Henderson 2007). We then performed a principal components analysis (PCA) to clarify redundancies and help indicating the variables to be either removed or maintained to compose the general linear models (GLMs). The selected geo-climatic variables were: for Axis 1, annual precipitation, precipitation of the coldest quarter, temperature annual range, potential evapotranspiration, soil water storage capacity, and soil fertility; for Axis 2, annual precipitation, aridity index, potential evapotranspiration, precipitation of the coldest quarter, soil fertility, soil storage capacity, soil texture, and temperature of the driest quarter; and for Axis 3, annual precipitation, annual mean temperature, temperature annual range, aridity index, potential evapotranspiration, precipitation seasonality, precipitation of the coldest quarter, precipitation of the driest quarter, soil fertility, soil storage capacity, soil texture, and temperature seasonality. All three GLMs were then free of multicollinearity.

We assessed the goodness-of-fit of GLMs through adjusted coefficients of determination (*R*^2^ adj.) and significance tests (*P* value) and examined the residuals graphically to check them for the assumption of linearity. We also tested the residuals for normality using D’Agostino-Pearson tests (Zar 2009), which failed the assumption for Axis 3 only. We only obtained normality for this axis after removing three areas (Maracás, Palmeiras, and Serra das Almas) identified as outliers based on studentized residuals. We then assessed the significance of each predictor variable on DCA scores through the total and partial coefficients of determination (*R*^2^) and significance tests (*P* value).

Because the lack of spatial independence may inflate the chance of committing Type I Errors (Legendre and Fortin 1989), we checked the residuals of GLMs for spatial structure (Diniz-Filho et al. 2003) through correlograms produced with Moran's I, as described above, and tested the global significance of spatial autocorrelation using the sequential Bonferroni correction (Fortin and Dale 2005). Because the spatial structure of GLM residuals was significant for both Axes 1 and 2 (i.e., at least one distance class showed a *P* value below the cutoff level), we modeled the spatial component applying the spatial eigenvector mapping method, SEVM (Dormann et al. 2007) on SAM 4.0, and included spatial filters to capture the remaining spatial structure. As spatial filters, we used vectors extracted from a truncated distance matrix (Borcard and Legendre 2002) with a truncation distance (*t*) defined as the distance connecting all points (areas) in the minimum spanning tree. After including one filter for Axis 1 and four filters for Axis 2 (all with significant correlation with the response variables), the residuals were all free of spatial structure.

### Differentiation of floristic units and their floristic relationships

After inspecting the ordination patterns of the 15 vegetation types, we observed that the altitudinal bands made no sense across the gradients summarized by ordination axes. We then eliminated this aspect and merged the 15 vegetation types into eight “SDTF floristic units” (Fig. 2) and gave them shorter names and acronyms: Crystalline Lowlands Caatinga (CrystallineCA), Sand Deposits Caatinga (SandCA), Rock Outcrops Caatinga (RockCA), Arboreal Caatinga (ArborealCA), Cerrado Dry Forest Enclaves (CerradoDF), Agreste and Brejo Dry Forests (AtlanticDF), Sand Deposits Dry Forest (SandDF), and Restinga Dry Forest (RestingaDF). We then performed, for heuristic purposes, two additional DCAs of selected subsets of areas to help clarifying the consistency of some proposed floristic units and the floristic relationship among them. They are treated here as DCA2 and DCA3, to contrast with the previous overall DCA (DCA1). DCA2 was performed with the areas of SandCA and CrystallineCA, because the two supposed floristic units were not clearly segregated from each other in the presence of the other units. As ordination patterns showed a clear floristic differentiation between the two units they were eventually maintained as distinct. DCA3 was performed with the purpose of clarifying the relationships of the ArborealCA with the three floristically closer SDTF units: the CrystallineCA, the CerradoDF, and the RockCA. The AtlanticDF and the poorly represented SandDF and RestingaDF were therefore eliminated of both DCA1 and 2. The geo-climatic variables with the strongest correlations with ordination scores were plotted as arrows on ordination diagrams (McCune and Grace 2002).

We then used one-way analyses of variance (ANOVAs) with the scores of floristic units in the first two axes of both DCA2 and DCA3 to assess their floristic differentiation across the gradients summarized in ordination axes. Even so, we followed some procedures beforehand. Firstly, we tested the spatial structure for the residuals of each ANOVA model (Moran's I coefficient) in SAM 4.0 (Rangel et al. 2010) applying sequential Bonferroni correction (Fortin and Dale 2005). When spatial independence failed, we included filters (SEVM modeling) as additional predictors, as described above, then performing a GLM. We also tested our ANOVA/GLM data for normality of residuals (D’Agostino-Pearson test). When normality failed, we removed outliers based on studentized residuals. When ANOVAs were significant, we applied post hoc Tukey test to assess variations among floristic units; for GLMs, we obtained 95% confidence intervals.

In order to assess the floristic links among floristic units, we obtained two additional matrices with the eight SDTF floristic units as row headings and the 1332 tree species as column headings. The entries of the first matrix were the species binary occurrence records and those of the second matrix were the species’ relative frequency across sample areas. We performed a cluster analysis of both matrices on PAST 1.93 (Hammer et al. 2001) using Sørensen (or Bray-Curtis) distance as dissimilarity measure and unweighted paired group as linkage method (McCune and Grace 2002). In order to characterize the tree flora of each floristic unit, we processed an indicator species analysis, ISA (Dufrêne and Legendre 1997), of the species matrix on PC-ORD 6.0. We then extracted the 10 species with highest frequencies across the samples areas of each floristic unit to compare them with the indicators produced by ISA. We also produced the species-area curve for each floristic unit using the bootstrap procedure in PCORD 6.0 and obtained the Chao2 estimator of species richness (Magurran 2004).

## Results

### Explanatory spatial and environmental variables underlying species composition variations

The eigenvalues obtained by DCA of the 186 SDTF areas (Table 1) were significant for the first three ordination axes. Nevertheless, as warned by McCune and Mefford (2011), we must bear in mind that “because *P* values for axes after Axis 1 depend on the results for Axis 1, these are presented for heuristic reasons only.” Despite this, Axis 2 and 3 were both poorly correlated with Axis 1 (*r* = 0.249 and 0.030, respectively) and both strongly orthogonal to it (93.8% and 99.9%, respectively), indicating a high level of mathematical independence. Axes 2 and 3 were also poorly correlated (*r* = −0.017) and strongly orthogonal (100.00%). The first eigenvalue itself approached that of a “strong” gradient (>0.5) in terms of species replacement (ter Braak 1995). In fact, this is confirmed by the lengths of gradients, which were all near three, because lengths between one and four represent a scale in species turnover from half-change to total replacement (Hill and Gauch 1980).

**Table 1:** Summaries of detrended correspondence analyses (DCAs) and respective permutation tests performed for binary occurrence records of 996 tree species in 186 areas of seasonally deciduous tropical forest (SDTF) of north-eastern and Central Brazil (DCA1) and two particular subsets extracting areas of selected Floristic Units (DCA2 and DCA3)

				Randomization tests	
				
DCAs	Axes	Eigenvalues	Length of gradients	*n*	P*
DCA1: 186 areas of seasonally dry tropical forest (SDTF)	1	0.45051	3.398	0	0.001
	2	0.32689	3.213	0	0.001
	3	0.20845	2.806	0	0.001
DCA2: 72 SDTF areas, of which 42 are CrystallineCA and 30 are SandCA	1	0.34703	2.733	0	0.001
	2	0.26428	2.404	0	0.001
	3	0.15892	2.488	0	0.001
DCA3: 125 SDTF areas, of which 42 are CrystallineCA, 20 are RockCA, 33 are ArborealCA and 31 are CerradoDF	1	0.50934	3.628	0	0.001
	2	0.21945	2.734	0	0.001
	3	0.15884	3.410	0	0.001

* *P* = (*n*+1)/(*N*+1), where *n* is the number of randomizations with an eigenvalue more than the observed eigenvalue for that axis; *N* = total number of randomizations.

**Figure 3:**
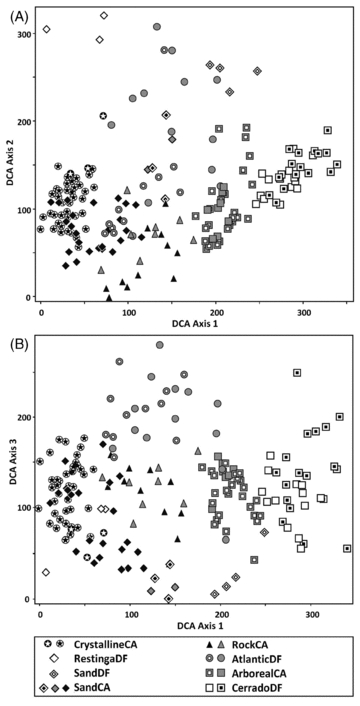
Diagrams showing the ordination of 186 areas of seasonally deciduous tropical forest (SDTF) of north-eastern and Central Brazil yielded by detrended correspondence analysis (DCA) of binary occurrence records of 996 tree species. Diagrams show ordinations on DCA axes 1 × 2 (A) and DCA axes 1 × 3 (B). The areas are discriminated by symbols corresponding to their vegetation types and respective SDTF floristic units (see Fig. 2 for details).

The ordination of areas by DCA1 (Fig. 3) segregated most a priori defined SDTF floristic units in distinct sectors of the diagrams. Axis 1 was very effective in segregating the areas of CerradoDF on the right side, immediately followed to the left by the areas of ArborealCA, and the areas of CrystallineCA, though these were mixed with some areas of SandCA and the three areas of RestingaDF (Fig. 3A and 3B). The areas of the other four floristic units were spread in between also mixed with areas of SandCA. Axis 2 segregated some of the remaining floristic units: the three areas of RestingaDF and four areas of SandDF were all placed on the top side, and the areas of RockCA, on the bottom. Axis 3 segregated an additional unit, containing the areas of AtlanticDF, on the top side, while most areas of SandCA and SandDF were concentrated on the bottom (Fig. 3B). The SandCA was therefore the less clearly segregated unit on all three axes. These patterns together with the geographical distribution of floristic units (Fig. 2) strongly suggest that both spatial and environmental factors underlie the floristic differentiation of SDTFs in the region.

**Figure 4:**
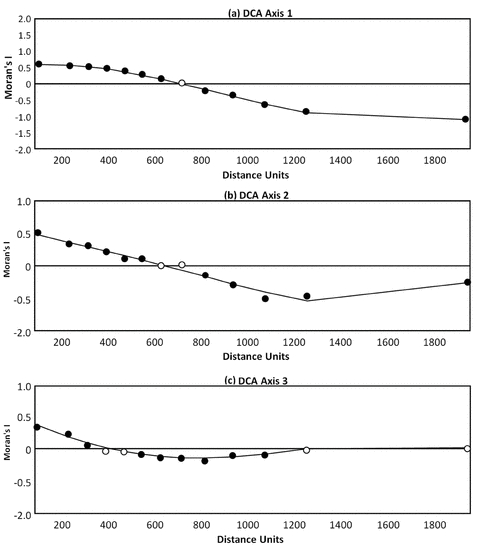
Correlograms produced for ordination scores of 186 SDTF areas in north-eastern and Central Brazil yielded by detrended correspondence analyses (DCA) of tree species binary occurrence records. Correlograms were produced for the first three ordination axis (Axis 1, 2, 3). Significant and nonsignificant Moran's I are shown as closed and open dots, respectively.

In fact, ordination scores were spatially structured in all three ordination axes (Fig. 4). The first two correlograms, produced for Axes 1 and 2, matched the “macroscale linear gradient” of Legendre and Fortin (1989), indicating a significant and positive spatial autocorrelation at shorter distance classes and a decline toward larger distance classes, becoming significantly negative above a certain distance class (Diniz-Filho et al. 2003). The third correlogram, produced for Axes 3, matched the “single thin bump” of Legendre and Fortin (1989), which behaves like the linear gradient pattern (i.e., positive followed by negative autocorrelation) up to a certain distance beyond which it becomes nonsignificant.

The three GLMs produced for the species composition gradients yielded by DCA1 are given in Table 2 together with the predictor variables used, both environmental and spatial. All three models produced strong correlations (0.9 > adjusted *R*^2^ > 0.7) and highly significant results (*P* < 0.01). The predictors with the strongest contribution to the model produced for Axis 1 were annual precipitation and soil water storage capacity, followed by annual temperature range, potential evapotranspiration, soil fertility, and Spatial Filter 1. A minor but significant contribution was given by precipitation of the coldest quarter. This set of variables is clearly related primarily with the regime of water availability in the substrate and secondarily with soil fertility. This is highly compatible with the contrasts between the Cerrado and Caatinga Domains in terms of climate and soils, the latter with its harsher water shortage periods and mineral-rich soils. This is also coherent with the discrimination by Axis 1 of the areas of CerradoDF and transitional ArborealCA on its right side while areas of core Caatinga SDTF units were spread toward the left side. The values of Spatial filter 1 increased toward the clump of CrystallineCA and SandCA. This was even clearer by plotting Filter 1 on the map of SDTF areas (see Appendix 1), which showed increasing spatial dependence from the outskirts to the core of the semiarid region.

**Table 2:** Results of the general linear models, GLMs, used to explain the floristic gradients summarized by the first three ordination axes yielded by detrended correspondence analysis (DCA1) of binary occurrence records of 996 tree species in 186 areas of seasonally deciduous tropical forest (SDTF) of north-eastern and Central Brazil. Contribution of each predictor variable or filter to the variance in ordination scores are given for each model and significant results are given in bold

Full models:	DCA1 Axis 1 *R*^2^ adj. = 86.18 *P* **< 0.01**			DCA1 Axis 2 *R*^2^ adj. = 78.02 *P* **< 0.01**			DCA1 Axis 3 *R*^2^ adj. = 72.56 *P* **< 0.01**		
			
Predictor variables	*R*^2^	Partial *R*^2^	*P*	*R*^2^	Partial *R*^2^	*P*	*R*^2^	Partial *R*^2^	*P*
Aridity index	–	–	–	0.02	0.05	0.78	0.43	0.80	0.25
Soil water storage capacity	19.30	36.87	**<0.01**	3.26	6.35	0.05	5.03	8.60	**0.01**
Soil texture	–	–	–	3.73	7.18	**<0.01**	6.48	10.82	**<0.01**
Soil fertility	3.94	10.65	**<0.01**	16.82	25.90	**<0.01**	2.94	5.21	**0.01**
Annual precipitation	21.97	39.94	**<0.01**	3.10	6.05	**<0.01**	0.60	1.11	0.18
Precipitation, driest quarter	1.23	3.60	**0.01**	2.99	5.84	**<0.01**	0.08	0.15	0.62
Precipitation, coldest quarter	–	–	–	–	–	–	0.10	0.20	0.57
Potential evapotranspiration	5.22	13.65	**<0.01**	0.44	0.90	0.22	8.32	13.47	**<0.01**
Precipitation seasonality	–	–	–	–	–	–	7.32	12.05	**<0.01**
Annual temperature	–	–	–	–	–	–	1.38	2.52	**0.04**
Temperature annual range	9.40	22.14	**<0.01**	0.17	0.35	0.45	1.64	2.97	**0.03**
Temperature, driest quarter	–	–	–	0.11	0.23	0.54	–	–	–
Temperature seasonality	–	–	–	–	–	–	1.11	2.03	0.07
Spatial filter 1	5.26	13.74	**<0.01**	10.52	17.94	**<0.01**	–	–	–
Spatial filter 2	–	–	–	4.81	9.08	**<0.01**	–	–	–
Spatial filter 4	–	–	–	3.89	7.48	**<0.01**	–	–	–
Spatial filter 6	–	–	–	1.53	3.08	**0.02**	–	–	–

As to the second model, the predictors with the highest contribution were, by far, soil fertility and Spatial Filter 1. Variables with lower but significant contribution were soil texture, annual precipitation, precipitation of the coldest quarter, and Spatial Filters 2, 4, and 6. Contrasting to the first model, the second gave a much stronger role to soil fertility than to ground water availability. In fact, Axis 2 did discriminate dry forest areas of sandy and nutrient poor soils occurring on seasonal climates, from areas of RockCA with their much richer soils but also much higher stress caused by water shortage. Spatial Filter 1 shows an additional pattern here, as it decreases toward the areas of RockCA appearing on the map (see Appendix 1) as increasingly negative values toward the Chapada Diamantina highlands. This pattern also appears when mapping Spatial Filters 2, 4, and 6 (Appendix 1), strongly suggesting that the RockCA is the least spatially structured of all Caatinga floristic units. As also evidenced on both maps and ordination diagrams, the latter filters also contributed to show additional spatial trends. Filter 2 shows increasing spatial dependence toward both the southwest (ArborealCA and CerradoDF) and northeast (northern CrystallineCA). Filter 4 also shows increasing spatial dependence toward the southwest but differs in its increasing values toward the central areas of SandCA. Filter 6 shows increasing spatial dependence toward the three units of Dry Forest. Spatial filters, to a great extent, captured the spatial structure of particular predefined SDTF units adding strength to their floristic consistency.

The strongest contributions to the third model were those of soil texture, potential evapotranspiration, precipitation seasonality, and soil water storage capacity. Soil fertility, annual temperature, and temperature annual range gave minor contributions. The chief role of soil texture is compatible with the discrimination of areas occurring on sandy substrates, which was much clearer on Axis 3 than on the former two. Likewise, Axis 3 yielded the best discrimination of AtlanticDF, which occur on transitional areas near the Atlantic forests or on hinterland moister highlands, where temperatures are lower and droughts are less severe.

### Consistency and characterization of proposed SDTF floristic units

**Figure 5:**
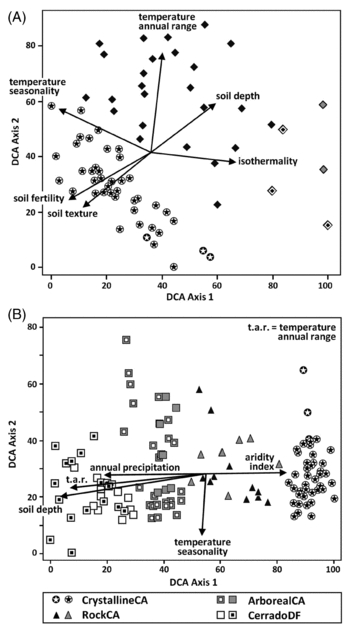
Diagrams showing the ordination of selected subsets of SDTF areas in north-eastern and Central Brazil yielded by detrended correspondence analyses (DCA) of tree species binary occurrence records; both showing ordinations on axes 1 × 2. (A) DCA1: 348 species occurring in 72 areas of CrystallineCA (42) and SandCA (30). (B) DCA2: 855 species occurring in 125 areas of CrystallineCA (42), RockCA (20), ArborealCA (33), and CerradoDF (31). The areas are discriminated by symbols corresponding to their vegetation types and respective SDTF floristic units (see Fig. 2 for details). Arrows represent correlations between geo-climatic variables and ordination scores.

**Figure 7:**
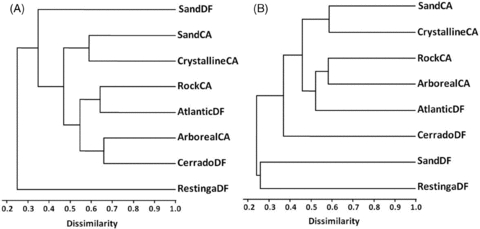
Dendrograms produced by cluster analyses of the tree species composition (A) and tree species relative frequency per area (B) in the eight SDTF floristic units of north-eastern and Central Brazil. The dissimilarity measure and linkage method were Sorensen (Bray and Curtis) distance and group average, respectively. Cophenetic coefficients were 0.8889, for species composition, and 0.8703 for species frequency.

The analyses above have already given strong evidence of floristic consistency for most SDTF units. On the other hand, they also indicated some drawbacks of the present dataset. Two supposed SDTF units, the RestingaDF and the SandDF, were poorly represented (three and four sample areas, respectively), though they were apparently consistent and highly differentiated. Where their removal did not contribute to clarify the remaining patterns they were maintained, as in DCA1 (Fig. 4) and cluster analyses (Fig. 7). Another drawback was that the high floristic heterogeneity and wide geographic range of the whole dataset probably contributed to blur the relevant but patchy floristic contrast between the CrystallineCA and the SandCA. Their respective areas were only clearly distinguished when processed in a separate analysis (DCA2) after removing all other units (Fig. 5A). SDTFs related to dystrophic sandy substrates are apparently very heterogeneous across the region, in coherence with their patchy geographic distribution, probably contributing to increase the total noise. This was clearly observed when the areas belonging to these units were removed from the dataset and the resulting diagram (DCA3) depicted a highly linear floristic gradient (Fig. 5B).

The eigenvalues of the first three ordination axes were all significant for both DCA2 and DCA3 (Table 1). The first eigenvalue of DCA3 represents a “strong” gradient (>0.5) in terms of species replacement (ter Braak 1995), while that of DCA2 represents a comparatively shorter gradient (>0.35). In both cases, the second and third axes were considerably shorter (0.15–0.26). Despite this, the lengths of gradients (all > 2.4) indicated that species replacement was above half-change in all axes and approached total replacement in Axes 1 and 3 of DCA3 (Hill and Gauch 1980). An explorative analysis using the graphic resources in PCORD 6.0 (Fig. 5A) indicated that the variables with the strongest correlations with the scores of DCA2 were temperature seasonality (*R* = −0.66), isothermality (*R* = 0.63), soil fertility (*R* = −0.62), soil texture (*R* = −0.57), and soil depth (*R* = 0.55), for Axis 1, and temperature annual range (*R* = 0.69) for Axis 2. The variables with the strongest correlations with the scores of DCA3 were soil depth (*R* = −0.85), temperature annual range (*R* = −0.73), annual precipitation (*R* = −0.72), and aridity index (*R* = 0.66) for Axis 1, and temperature seasonality for Axis 2 (*R* = −0.57).

Ordination scores produced by DCA2 differed significantly between the areas of CrystallineCA and SandCA for both Axis 1 (multiple adjusted *R*^2^ = 0.708; *F* = 28.422; *P* < 0.0001) and Axis 2 (multiple adjusted *R*^2^ = 0.836; *F* = 34.025; *P* < 0.0001) in GLMs that incorporated five and 10 spatial filters for Axis 1 and 2, respectively, and eliminated three outliers (Ubajara, carrasco; Novo Oriente carrasco; and Frei Paulo) for Axis 1 only. The means of area scores were significantly higher for SandCA than for CrystallineCA, using 95% confidence intervals, on both Axis 1 (*F*_1,62_ = 39.321; *P* < 0.0001) and Axis 2 (*F*_1,60_ = 60.384; *P* < 0.0001).

Ordination scores produced by DCA3 differed significantly among the areas of the four floristic units for Axis 1 only (multiple adjusted *R*^2^ = 0.955; *F* = 661.679; *P* < 0.0001) in a GLM that incorporated one spatial filter. Score means differed significantly among all four floristic units using both 95% confidence intervals (*F*_3,120_ = 381.87; *P* < 0.0001) and post hoc Tukey tests (CrystallineCA > RockCA > ArborealCA > CerradoDF).

**Figure 6:**
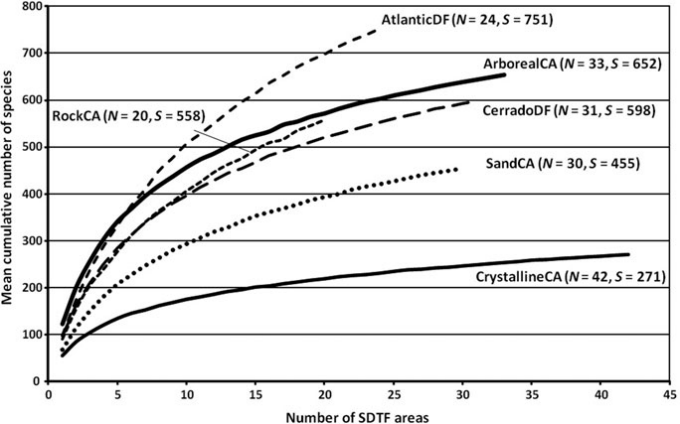
Mean cumulative number of species per number of sample areas in six SDTF floristic units of north-eastern and Central Brazil. *N* = number of areas; *S* = number of species; mean curves were obtained through 500 repeated subsamplings for each subsample size.

There was strong evidence that the species richest floristic units were the AtlanticDF and the RockCA. Despite their comparatively smaller sample size, the slopes of their species-area curves were both very steep all over (Fig. 6) and they also produced the highest estimated species richness: Chao2 = 1060 and 881 species, respectively. Immediately below, the ArborealCA (Chao2 = 787 species) and the CerradoDF (Chao2 = 747 species) also produced species-area curves with a slight slope decline at larger sample sizes (Fig. 6). This slope decline was more pronounced for the SandCA and the CrystallineCA, both with much lower estimated species richness: Chao2 = 587 and 342 species, respectively. The estimated species richness of the two poorly sampled floristic units, the SandDF and RestingaDF, were Chao2 = 457 and 251 species, respectively.

The eight floristic units shared a considerable number of species, as 876 of the 1332 species of the dataset (65.8%) were recorded in two or more units. That left 456 singletons (34.2%), 141 of which occurred in the AtlanticDF, 119 in the CerradoDF, 61 in the ArborealCA, 45 in the SandDF, 34 in the RestingaDF, 28 in the RockCA, 22 in the SandCA, and only six in the CrystallineCA. Only 13 species were recorded in all floristic units: *Myracrodruon urundeuva*, *Handroanthus impetiginosus*, *Cereus jamacaru*, *Combretum leprosum*, *Croton blanchetianus*, *Anadenanthera colubrina*, *Senegalia polyphylla*, *Senna spectabilis*, *Brosimum gaudichaudii*, *Maclura tinctoria*, *Eugenia punicifolia*, *Talisia esculenta,* and *Ximenia americana* (see Appendix 2 for species authorities).

Despite the large proportion of species shared by different floristic units, their relative frequency across sample areas varied widely among them, so that the ISA and associated Monte Carlo tests could discriminate 537 species as indicators of one of the six main floristic units (the two poorly sampled units were eliminated). See Appendix 2 for ISA results, including indicator species and their relative frequencies in each floristic unit. Ten additional species, unrelated by Monte Carlo tests to any floristic unit, are also included there because of their high frequency across sample areas (>50% in two or more units): *Spondias tuberosa*, *H. impetiginosus*, *Pilosocereus pachycladus*, *Neocalyptrocalyx longifolium*, *Maytenus rigida*, *C. leprosum*, *Amburana cearensis*, *Chloroleucon foliolosum*, *E. punicifolia,* and *Tocoyena formosa*.

The two poorly sampled floristic units, the SandDF and the RestingaDF, were both strongly segregated from the other six in the cluster analyses performed with the floristic units (Fig. 7), and are therefore treated here as outliers. In the dendrogram produced for species composition (Fig. 7A), the six remaining units were firstly coupled into three clusters: (1) ArborealCA and CerradoDF, (2) RockCA and AtlanticDF, and (3) CrystallineCA and SandCA. Above that, groups (1) and (2) formed a new cluster before merging with (3). In the dendrogram produced for species frequencies across sample areas (Fig. 7B), the outstanding contrast was the segregation of the CerradoDF from the other five floristic units, all part of the Caatinga Domain and its peripheral areas. Contrasting to the previous case, here the ArborealCA clustered first with the RockCA, and then this group clustered with the AtlanticDF. Only then this main group clustered with that formed by the CrystallineCA and SandCA. The ArborealCA, therefore, has a stronger compositional floristic link with the CerradoDF, but a stronger floristic link with the RockCA and the AtlanticDF in terms of most frequent species across sample areas.

## Discussion

### Spatial and environmental variables underlying species distributions and floristic units

The first and second hypotheses were not rejected, that is variations in tree species composition across SDTF areas in the region did show both spatial structure and significant relationships with environmental variables, and eight SDTF floristic units could be recognized in the region based on coherent and peculiar species composition and geo-climatic features. In fact, as usual in Neotropical SDTFs (see Pennington et al. 2009), there were a few widespread species and many endemic to particular sets of floristic units causing high β-diversity in the regional SDTFs, a strong indication that the whole system is quite stable and highly dispersal limited. This is of particular importance within the Caatinga Domain where most floristic units are contiguous and not fragmented, as those of the Cerrado Domain (CerradoDFs), and the effect of spatial proximity may not be obscured by spatial isolation. This brings additional evidence that successful immigration of a great number of species across Neotropical SDTFs is strongly limited by niche-based controls (Pennington et al. 2009). This also brings a new light to the fact that the Caatinga Nucleus outstands for its high proportion of endemics, ca. 34% of the woody flora (Leal et al. 2005), while most nonendemics, ca. 60%, are shared with other SDTF nuclei (Giulietti et al. 2002). The Caatinga Domain is therefore a heterogeneous mosaic of eco-regions and vegetation types, and this is an important aspect for conservation efforts (Velloso et al. 2002; Queiroz 2006). The positive spatial autocorrelation toward floristic and ecologically differentiated eco-regions are indication of their uniqueness and, therefore, high conservation value. On the other hand, the negative spatial autocorrelation toward the Chapada Diamantina is a strong indication of increased floristic and ecological heterogeneity, probably related to the usually high environmental patchiness of highlands, another feature leading to increased conservation value.

Two proposed SDTF floristic units, the RestingaDF and the SandDF, were poorly sampled and are better left for future confirmation regarding their consistency. Three of the six remaining units; the CrystallineCA, the SandCA, and the CerradoDF; are already quite consolidated entities in the literature for their ecological and floristic consistency and most of their indicator species (see Appendix 2) have already being cited as such (e.g., Oliveira-Filho and Ratter 2002; Prado 2003; Queiroz 2006; Oliveira-Filho et al. 2006; Cardoso and Queiroz 2010). The novelty then is the floristic and environmental characterization of the AtlanticDF, RockCA, and ArborealCA. The former clearly contains a great proportion of species shared with typical (and moister) Atlantic forests, such as *Albizia polycephala*, *Clusia nemorosa*, *Tabernaemontana solanifolia*, *Myrcia sylvatica*, *Psidium brownianum*, *Coutarea hexandra, Byrsonima sericea*, *Zanthoxylum rhoifolium*, *Cordia superba*, *Myrsine guianensis*, *Roupala paulensis*, *Manilkara rufula,* and *Miconia albicans*. The AtlanticDF may be then considered as a transition to the Atlantic Forest Domain. Likewise, the ArborealCA may be considered as a transition to the Cerrado Domain because of the high frequency of widespread species shared by both floras. The RockCA, instead, is not only a consistent floristic unit but a newly proposed type of *caatinga* vegetation because it is characterized by a particular set of species (see Appendix 2) concentrated in four families: Fabaceae (e.g., *Blanchetiodendron blanchetii*, *Mimosa irrigua*, *Poincianella laxiflora*, *Pseudopiptadenia brenanii*, *Pterocarpus villosus*, *P. zehntneri*, and *Pterodon abruptus*), Cactaceae (e.g., *Brasilicereus phaeacanthus*, *C. albicaulis*, *Facheiroa squamosa*, *Pereskia aureiflora*, *P. bahiensis*, *P. stenantha*, *P. glaucochrous*, *P. pentaedrophorus*, *Stephanocereus leucostele* (Cactaceae), Euphorbiaceae (e.g., *Cnidoscolus bahianus*, *C. argyrophylloides*, and *Jatropha palmatifolia*), and Rutaceae (*Galipea ciliata* and *Pilocarpus sulcatus*). Such species are also found at lower frequencies in the ArborealCA and AtlanticCA, therefore explaining their stronger links with the RockCA and the transitional nature of all three floristic units, which occur precisely where the levels of aridity are lessened. The floristic blend and lessened aridity probably explain much of the higher species richness of these three transitional floristic units compared to the much poorer flora of the semiarid CrystallineCA and SandCA. The CerradoDF is also particularly rich in species probably because of the great contribution of species shared with the floras of both the *cerrado* and gallery forests (in fact, all selected indicators).

The distribution patterns of the SDTF tree flora in the region is primarily related with interacting geo-climatic variables that determine the availability of ground water across time, and secondarily with those related to the amount of mineral nutrients in the substrate (Tables 3 and 4, and see Appendix 3 for a key to SDTF units). This is no surprise, as light and water are undisputedly the leading resources determining distribution patterns of terrestrial plants worldwide and, generally speaking, where water deficits are the main cause of stress, competition for light among plants is relaxed because shading by plant mass is reduced (Huggett 1995). More than that, the intense solar radiation in arid and semiarid habitats commonly aggravates water deficits because it increases both temperature and evapotranspiration. Figures of mean annual temperatures of the Caatinga Domain (26–28°C) rank indeed among the highest in Brazil (Cardoso and Queiroz 2010).

**Table 3:** Climatic variables (means ± standard deviations) in the *N* areas of each SDTF floristic unit, which contributed significantly to the general linear models, GLMs, used to explain the floristic gradients summarized by the first three DCA1 axes (see Table 2)

		Precipitation				Temperature	
				
Floristic units	*N*	Annual (mm)	Driest qt. (mm)	Seasonality (%)	Potential evapotranspiration (mm)	Annual (°C)	Annual range (°C)
CrystallineCA	42	718 ± 225	46 ± 62	87 ± 25	1628 ± 119	24.5 ± 1.8	13.6 ± 1.5
RestingaDF	3	1287 ± 216	51 ± 13	78 ± 9	1648 ± 39	26.1 ± 0.6	10.0 ± 3.3
SandDF	4	1118 ± 278	37 ± 23	94 ± 12	1896 ± 18	26.3 ± 0.9	16.1 ± 0.6
SandCA	30	635 ± 149	37 ± 51	85 ± 21	1790 ± 146	24.6 ± 1.1	16.0 ± 1.5
RockCA	20	765 ± 174	31 ± 30	82 ± 16	1710 ± 131	23.6 ± 1.4	16.6 ± 1.6
AtlanticDF	24	763 ± 119	91 ± 51	55 ± 19	1521 ± 125	22.1 ± 1.3	13.7 ± 1.7
ArborealCA	33	875 ± 72	8 ± 7	98 ± 4	1802 ± 84	23.9 ± 0.8	18.4 ± 1.4
CerradoDF	31	1272 ± 206	21 ± 11	90 ± 6	1694 ± 101	23.0 ± 1.7	17.9 ± 2.2

qt. = quarter.

**Table 4:** Substrate-related variables (first quartile/median/third quartile) in the *N* areas of each SDTF floristic unit. All but soil depth contributed significantly to the general linear models, GLMs, used to explain the floristic gradients summarized by the first three DCA1 axes (see Table 2). Medians are enhanced in bold

Floristic units	*N*	Soil fertility^1^	Soil texture^2^	Soil depth^3^	Soil water storage^4^
CrystallineCA	42	2.25/**3**/3	3/**3**/3	1/**1**/1	3/**3**/3
RestingaDF	3	1/**1**/1	1.5/**2**/2	3/**3**/3	4.5/**6**/6
SandDF	4	1/**1**/1	2/**2**/2	2/**2**/2	4/**4**/4
SandCA	30	1/**1**/1	1/**1**/1.75	1/**2**/3	2/**3**/3
RockCA	20	2/**3**/3	2/**2.5**/3	1/**1**/1	2/**2.5**/3
AtlanticDF	24	1/**2**/2	3/**3**/3	2/**2**/2	3.75/**6**/6
ArborealCA	33	3/**3**/3	3/**3**/3	2/**2**/3	6/**6**/9
CerradoDF	31	2/**2**/3	3/**3**/3	3/**3**/3	9/**9**/9

1 Soil fertility: 1 = “dystrophic” (saturation of bases < 30%), 2 = “mesotrophic” (30–70%), 3 = “eutrophic” (>70%).

2 Soil texture: 1 = “sandy” (sand fraction > 70%), 2 = “sand-loamy” (30–70%), 3 = “loamy” (<30%).

3 Soil depth: 1 = “bare rock to shallow” (0–10 cm deep), 2 = “deep to shallow” (10–50 cm), 3 = “deep” (>50 cm)

4 Soil water storage capacity = soil texture × soil depth.

As a primary source of water, rainfall regime is a chief factor determining the present distribution of SDTFs (Gentry 1995). In fact, the mean annual precipitation was above 1000 mm (see Table 3) only for SDTF areas situated beyond the outskirts of the Caatinga Domain, either in the Atlantic Forest (those of RestingaDF) or the Cerrado Domains (those of SandDF and CerradoDF). The vast majority of areas within the Caatinga Domain (those of CrystallineCA, SandCA, and RockCA) had mean annual precipitations below 800 mm and this main split corresponds to that between “moist seasonal climates,” with regular rainy and dry seasons, and “semiarid seasonal climates,” with more erratic and much shorter rainfall seasons (Rodal et al. 2008; Cardoso and Queiroz 2010). The rainfall regime of the areas of ArborealCA was typically transitional between these two, with intermediate figures for mean annual precipitation (875 ± 72 mm SD) and the highest and lowest figures for precipitation seasonality and precipitation of the driest quarter, respectively. They are therefore treated here as “strongly seasonal climates.” In great contrast, the rainfall regime of the transitional areas between the Caatinga and the Atlantic Forest Domains, that is those of AtlanticDF (see Mendes et al. 2010), is characterized by the lowest and highest figures for precipitation seasonality and precipitation of the driest quarter, respectively. They are therefore treated here as “semiarid aseasonal climates.” The widely known “monsoon” pattern of increasing seasonality away from continental coastlands (Lomolino et al. 2010) is certainly the main factor involved in the two contrasting transitional climates and floras of the Caatinga outskirts, that is, the western strongly seasonal and the eastern semiarid aseasonal climates. Continentality also affects temperature annual range and potential evapotranspiration, both much lower in areas lying much closer to the ocean, as those of AtlanticDF and RestingaDF, than in inward continental areas (Table 3).

On the other hand, rainfall regime can only explain the distribution of SDTFs, as well as their floristic and physiognomic variations, when other interacting factors are taken into consideration. As most plants are able to capture ground water only, its availability to the root system is highly affected by substrate-related factors; particularly landform, soil depth, and soil texture (Gentry and Emmons 1987; Araújo et al. 1995; Queiroz 2006); all of which were key factors segregating most floristic units in the study region (see Table 4). In a larger scale, landform operates on climatic shifts caused by the intermission of highlands within the Caatinga Domain where temperatures drop and rainfall is locally increased by the forced uplift of air masses on the windward slopes and decreased on the leeward side (Rodal 2002; Ferraz et al. 2004; Araújo et al. 2011). On the other hand, elevations rising above the crystalline basement include not only massive ridges and tablelands, as the Chapada Diamantina and Serra do Araripe, but also several extruding granitic inselbergs and limestone outcrops of various sizes, which are scattered all over the Caatinga Domain (Cardoso and Queiroz 2010). In addition to the local climatic heterogeneity caused by the elevations themselves, there is also the heterogeneity of the substrate caused by the wide variation of bedrocks, which includes quartzite, sandstone, limestone, and granite, and therefore gives rise to a wide array of soil types. The substrate has already being recognized as a leading factor determining the distribution of many plant species within the Caatinga Domain, particularly among Cactaceae (Taylor and Zappi 2004) and Fabaceae (Queiroz 2006; Cardoso and Queiroz 2010). This overall high substrate heterogeneity probably explains the weak spatial dependency among the areas of RockCA and strongly suggests that further studies may decompose its flora and habitats into more refined SDTF floristic units.

In a smaller scale, landforms may operate on the heterogeneity of ground water distribution. In general, bare rocks, shallow soils, and steep sloping areas tend to be strongly drained and therefore hold very short water stocks. On the other hand, sedimentary valleys and depressions tend to accumulate drainage water and may undergo temporary floods when water drainage is obstructed (Cardoso and Queiroz 2010). This includes not only long-lasting underground water stocks, such as those along intermittent rivulets, but also wide flatlands that are ordinarily flooded during the rainy periods, all commonly indicated by evergreen trees, such as *Ziziphus joazeiro*, *Colycodendron yco*, *Geoffroea spinosa*, *Copernicia prunifera*, *Erythrina velutina,* and *Licania rigida* (Prado 2003). In some areas of ArborealCA, in northern Minas Gerais, there are also smaller depressions formed by collapsed and packed limestone layers, the so-called “furados,” which are also liable to floods.

Soil mineral fertility within the Caatinga Domain is strongly associated with soil texture as it is much lower in sand deposits than in any other substrate (see Table 4). Soil fertility is also the leading factor explaining the occurrence of the Dry Forest Enclaves within the Cerrado Domain, where rainy seasons are generally longer and most soils are poor in mineral nutrients and covered by savannas, seasonal marshes or gallery forests. The SDTFs of the Cerrado Domain are therefore restricted to patches or mineral-rich soils, particularly those formed from either limestone outcrops, as in the Paranã River Valley (Scariot and Sevilha 2005), or basalt outflows, as in the Mato Grosso de Goiás (Oliveira-Filho and Ratter 2002; see Fig. 2). Deciduousness in seasonal climates is a more effective plant strategy to endure periods of water deficit where soils are richer in mineral nutrients and therefore more favorable to leaf regrowth in the rainy season (Oliveira-Filho et al. 2006). In great contrast, where soils are poor in minerals, as in sand deposits, deciduousness is a less effective strategy to face water shortage and leaves are commonly produced year-round, with no clear seasonality (Rocha et al. 2004). The three SDTF floristic units found on sandy and dystrophic substrates may rely on alternative strategies as well as on long-lasting ground water stocks. The SandDF is highly coincident with the Campo Maior Complex, one of eight eco-regions proposed by Velloso et al. (2002) for the Caatinga, and not for the Cerrado Domain, as proposed here, because of its moist seasonal climate.

In the semiarid seasonal climates of the Caatinga Domain, however, the dystrophic and sandy substrate probably represents an additional stress to the periods of water shortage. Velloso et al. (2002) treated these areas as three main disjunct eco-units, the Ibiapaba-Araripe highlands, in the northwest, the Raso da Catarina depression, in the east, and the São Francisco dunes, in the west, as they do show some floristic, ecological, and environmental peculiarities (Araújo et al. 2005). On the other hand, there was also evidence that these *caatingas* of sandy and dystrophic soils formed a main floristic unit contrasting with the predominant *caatinga* on shallow and richer soils of the crystalline basement (Rodal and Sampaio 2002). Queiroz (2006) eventually proposed that the Caatinga comprises two main distinct floras, one associated with the crystalline basement and another with the sand sediments, and that this latter harbors most of the endemic and ancient Caatinga flora. The author argued that the present restricted and disjunct distribution of the SandCA would result of the process of geological pediplanation that exposed the crystalline surfaces during the Late Tertiary and early Quaternary (Ab'Sáber 1974). This process would result in the establishment of species typical of other SDTF nuclei on the recently exposed crystalline lowlands while a great proportion of the original flora would become confined to sand deposits. An evidence for this was given by the tree flora of the SandCA, which was significantly richer in species than that of the CrystallineCA, despite its considerably smaller geographic extent and number of sampled areas. The number of species singletons was also ca. four times higher in the Sand Deposits than in the CrystallineCA (22 × 6), suggesting a higher level of endemism, as stressed by Cardoso and Queiroz (2010). In addition to this, the CrystallineCA forms a clear continuous floristic gradient extending to the RockCA, ArborealCA, and CerradoDF, while the SandCA shows no clear connection with this gradient. The distinction between the CrystallineCA and SandCA became clear only after removing the other SDTF areas and this strongly suggests that the former is actually a blend of species originated from two contrasting sources, the ancient autochthonous flora of semiarid sand deposits and the immigrant alien flora of seasonally dry forests. The blend was probably made up of an extraction from both sources of species that coped better with the new and expanding habitat. This was probably quite an unstable process, as there is strong evidence that the rainfall regime of the Crystalline basement was heterogeneous both in space and time during the climatic fluctuations of the Quaternary (de Oliveira et al. 2005; Mayle 2006). If so, the Sand Deposits were probably more stable in terms of maintaining the primitive xeric flora because of its low water storage capacity.

### Floristic and environmental features of the Arboreal Caatinga

The third hypothesis was confirmed only within the frame of the second hypothesis, that is, the ArborealCA is a consistent floristic unit with particular environmental features. Nevertheless, the proposal of Andrade-Lima (1971) of a stronger floristic link with the “typical *caatinga*” vegetation than with outlying deciduous forests was only partially confirmed. In terms of species composition, the ArborealCA was closest to the CerradoDF, indicated by the highest Jaccard's index, 49.3%, in all paired comparisons between the six main floristic units. On the other hand, if we consider the CrystallineCA as the “typical *caatinga*,” we find the lowest Jaccard's index, 22.9%, in all comparisons with the ArborealCA. The ArborealCA was actually much closer to the “a-typical” RockCA (47.2%), AtlanticDF (40.4%), and SandCA (38.6%). This whole group though apparently bridges two extremes of floristic dissimilarity, the CrystallineCA and the CerradoDF, which produced the lowest of all Jaccard's index, 17.0%. In terms of species frequency across sample areas, however, the ArborealCA was closest to the RockCA (ED^2^ = 188,500), farther from the CerradoDF (ED^2^ = 210,900), but still far from the CrystallineCA (ED^2^ = 359,600). This reinforces a view of the ArborealCA as a unique SDTF floristic unit with a peculiar strongly seasonal climate, characterized by a low but more predictable annual rainfall. The transitional character of both its flora and environment is, at least, arguable. As happens to many descriptive tools, transitions may be deceitful when they fail to include relevant aspects of abstracted reality.

The common identity of the ArborealCA with other SDTF units of the Brazilian Group (sensu Linares-Palomino et al. 2011) is clear from its long periods of water shortage and the high frequency of widespread SDTF species, such as *A. colubrina*, *M. urundeuva*, *H. impetiginosus*, *Aspidosperma pyrifolium*, *S. polyphylla, Piptadenia viridiflora*, *Bauhinia acuruana*, *A. cearensis*, *Schinus brasiliensis*, *Annona leptopetala*, *C. trichotoma*, and *Platymiscium floribundum*. Certainly because of this, the identity of the ArborealCA has been controversial for a long time. Some authors, such as Rizzini (1979) and Veloso et al. (1991), have placed them together with the seasonally deciduous forests of the Cerrado Domain within the single identity of “mata seca” ( = dry forest). Others suggested that the whole set of seasonally deciduous forests should be considered as part of the wide concept of Atlantic Forests and therefore the “driest” expression of a single vegetation *continuum* determined by increasing rainfall seasonality (Oliveira-Filho and Fontes 2000; Amorim et al. 2005; Oliveira-Filho et al. 2006). This ended up in the incorporation by the Brazilian Government of the ArborealCA within the official circumscription of the Atlantic Forest Biome (IBGE 2008). On the other hand, Luetzelburg (1922), Magalhães (1961), Andrade-Lima (1964, 1975), and Cardoso and Queiroz (2010), based on physiognomy and composition, recognized the ArborealCA (*caatinga arbórea*) as the forested expression of the *caatinga* vegetation. In fact, according to Andrade-Lima (1975), the ArborealCA is what was originally referred to as *caatinga* in the Tupi language.

In fact, this first formal assessment of the floristic identity of the ArborealCA demonstrates that the three views above are all different, and valid, perspectives. In the present case, we distinguished a set of adjacent SDTF floristic units based on distinct tree species composition. Despite this, a large proportion is shared by two or more units and, at the same time, each unit has its particular set of indicators, that is, species that are significantly more frequent across the sample areas of that particular unit. Using the ArborealCA as an example, 44% of the species registered in this unit are catalogued as widespread in the whole Caatinga Domain (based on Giulietti and Forero 1990; Rodal et al. 2008; and Cardoso and Queiroz 2010). On the other hand, species typical of the Atlantic forest are found in moister sites within the ArborealCA, such as *Agonandra excelsa*, *Chrysophyllum marginatum*, *Casearia lasiophylla*, *Dendropanax cuneatus*, *Dictyoloma vandellianum,* and *Protium spruceanum*. Despite this, there is a large group that is disproportionately more frequent in areas of ArborealCA, including *C. selloana*, *Cyrtocarpa caatingae*, *Leucochloron limae*, *P. bahiensis*, *Quiabentia zehntneri*, *Pseudobombax simplicifolium*, *Copaifera magnifolia*, *Attalea vitrivir*, *Acosmium diffusissimum*, *Piranhea securinega*, *Riedeliella graciliflora*, *Stillingia saxatilis*, *Tabebuia reticulata*, *B. blanchetii*, *Goniorrhachis marginata*, *Tabaroa caatingicola,* and *P. pluviosa*. Similarly, species typical of the ArborealCA may also occur elsewhere, as happens to *Cecropia saxatilis*, *Ceiba rubriflora*, *Commiphora leptophloeus*, and *C. jamacaru* that may occur in CerradoDFs on shallow limestone outcrops.

### Concluding remarks

The present study brings a relevant contribution to the current description of SDTFs as essentially a metacommunity where much of the species composition and abundance distribution in each fragment is largely determined by ecological drift, that is neutral dynamics governed by demographic stochasticity (e.g., Pennington et al. 2009, Cardoso and Queiroz 2010). We here provide evidence that there is no ground for a radical exclusion of niche-based dynamics among species as an underlying force driving both the species richness and dominance in SDTFs because of the clear spatial and environmental partition among a considerable number of species (Weiher et al. 2011). As most floristic units of the present study are large and adjacent, they are more open to species exchange and exposed to stronger migration pressures. The balance between stochastic and deterministic forces depends on the size of the species pool; the larger the size the more competitors will vary in their degree of competitive asymmetry (Orrock and Watling 2010). The opposite would occur in smaller and isolated areas where competitors become effectively neutral, but even in these cases there is also propagule pressure from adjacent alien species pools. This explains why typical *cerrado* and SDTF species form a peculiar blend in CerradoDF, and this may contribute to increase competitive asymmetry in a different manner. The balance between deterministic and stochastic processes may also vary across ecological gradients depending on disturbance levels, primary productivity and biotic interactions (Chase and Myers 2011; Weiher et al. 2011), which is precisely the case of the floristic units of the present study. As a final statement, we strongly emphasize that the floristic SDTF units should be given the status of eco-regions to help driving the conservation policy regarding the protection of their biodiversity.

## Supporting Information

**Appendix 1.** Spatial filters plotted as bubbles on maps of SDTF areas (see Fig. 2).

**Appendix 2.** Species recorded with frequency > 50% inat least one of the six main floristic units of seasonallydeciduous tropical forest (SDTF) of north-eastern and CentralBrazil or significantly related to a floristic unit by indicatorspecies analysis (ISA) and associated Monte Carlo permutation tests Figures are species' frequencies in each floristic unit and symbolsare significance levels: **P* < 0.05,^1^*P* < 0.01,^1/2^*P* <0.005,^§^*P* < 0.001.

**Appendix 3.** A key to the eight SDTF units of north-eastern and Central Brazil based on both Tables 3 and 4 with the 25 most frequent indicator species of the six main units (see full list in Appendix 2).
