# Supplementary figures and images for: Identity and relationships of the Arboreal Caatinga among other floristic units of seasonally dry tropical forests (SDTFs) of north-eastern and Central Brazil
Source: Ecol Evol. 2012 Feb;2(2):409–28. doi: 10.1002/ece3.91 (PMC3298952; doi:10.1002/ece3.91)

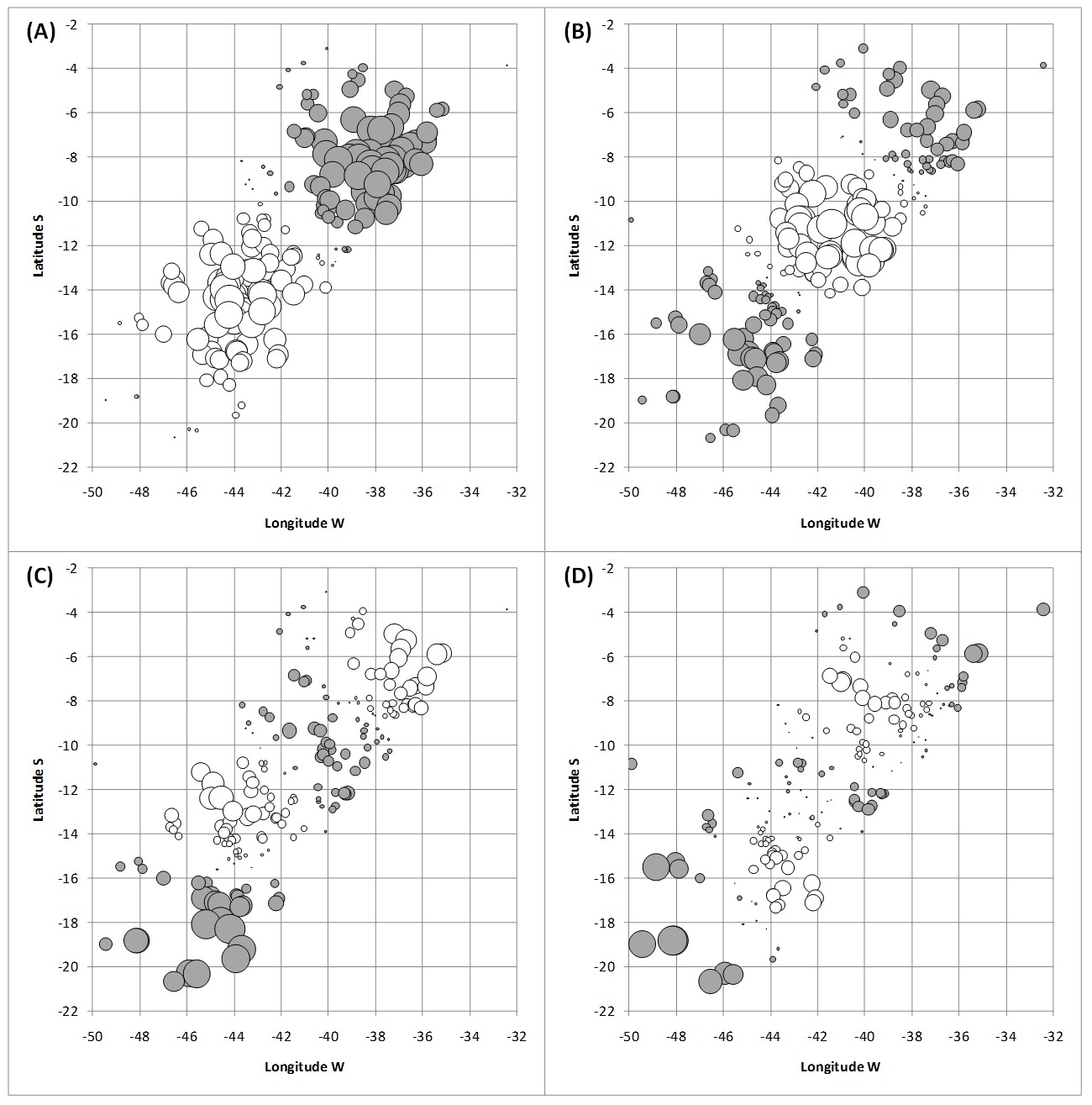

Supplement: Supplementary file 1 [file ece30002-0409-SD1.jpg]

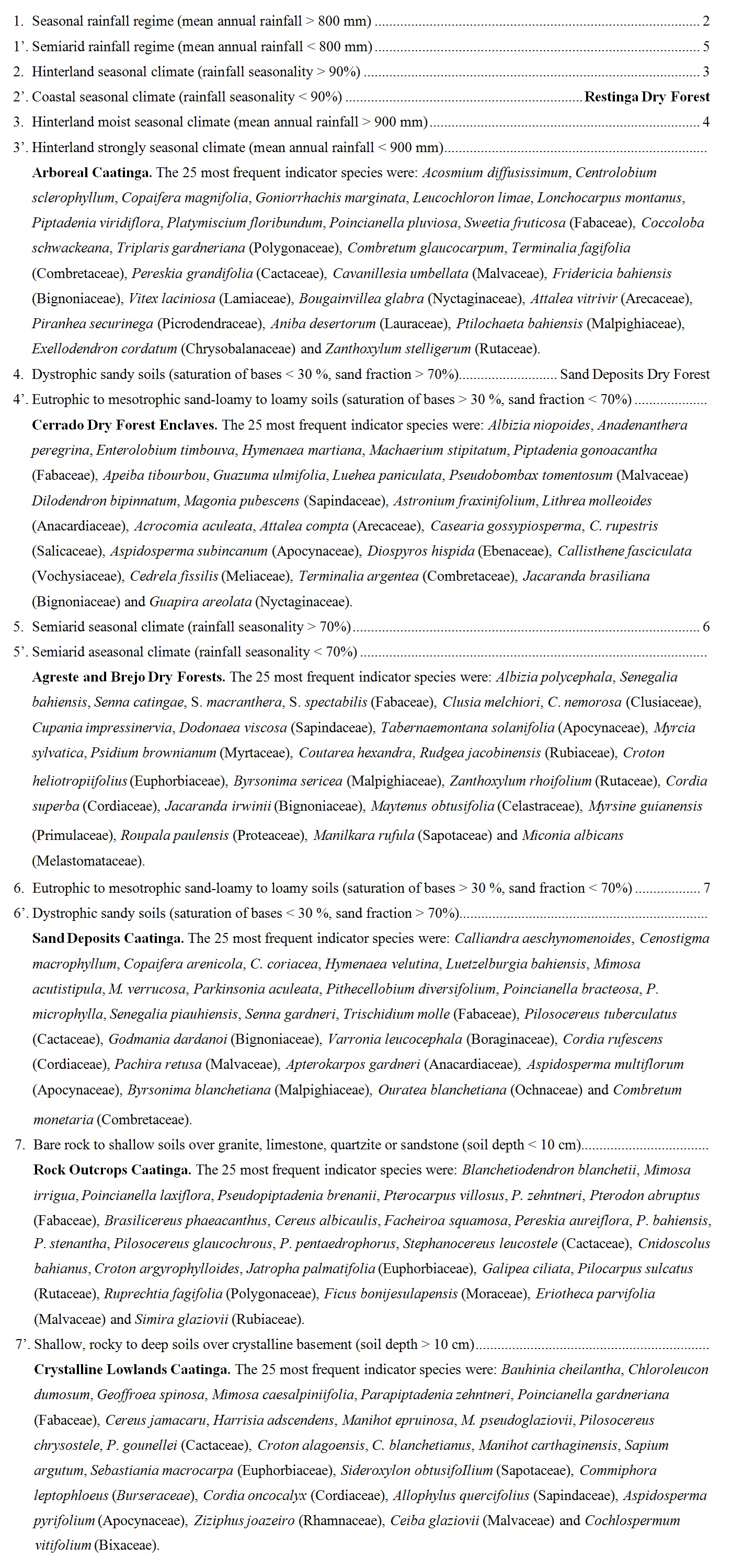

Supplement: Supplementary file 3 [file ece30002-0409-SD3.jpg]
